# Exposure of Larvae of the Solitary Bee *Osmia bicornis* to the Honey Bee Pathogen *Nosema ceranae* Affects Life History

**DOI:** 10.3390/insects10110380

**Published:** 2019-10-31

**Authors:** Kathrin Bramke, Uta Müller, Dino P. McMahon, Jens Rolff

**Affiliations:** 1 Institut für Biologie, Universität Berlin, 14195 Berlin, Germany; dino-peter.mcmahon@bam.de (D.P.M.);; 2 Abteilung 4 Material und Umwelt, Bundesanstalt für Materialforschung und-prüfung (BAM), 12205 Berlin, Germany

**Keywords:** wild bees, *Nosema ceranae*, *Osmia bicornis*, pathogen transmission, solitary bees, bee health, bee diseases

## Abstract

Wild bees are important pollinators of wild plants and agricultural crops and they are threatened by several environmental stressors including emerging pathogens. Honey bees have been suggested as a potential source of pathogen spillover. One prevalent pathogen that has recently emerged as a honey bee disease is the microsporidian *Nosema ceranae*. While the impacts of *N. ceranae* in honey bees are well documented, virtually nothing is known about its effects in solitary wild bees. The solitary mason bee *Osmia bicornis* is a common pollinator in orchards and amenable to commercial management. Here, we experimentally exposed larvae of *O. bicornis* to food contaminated with *N. ceranae* and document spore presence during larval development. We measured mortality, growth parameters, and timing of pupation in a semi-field experiment. Hatched individuals were assessed for physiological state including fat body mass, wing muscle mass, and body size. We recorded higher mortality in the viable-spore-exposed group but could only detect a low number of spores among the individuals of this treatment. Viable-spore-treated individuals with higher head capsule width had a delayed pupation start. No impact on the physiological status could be detected in hatched imagines. Although we did not find overt evidence of *O. bicornis* infection, our findings indicate that exposure of larvae to viable *N. ceranae* spores could affect bee development.

## 1. Introduction

Pollination provided by wild and domesticated bees is essential for wild flowers and agricultural crops. Biodiversity and food security rely on pollinator communities [1,2,3]. Bee population declines have been reported globally [2,4,5,6,7,8,9,10,11,12,13]. Causes for decline include land use changes, habitat loss, and fragmentation [14], pesticide use [15,16,17], pathogens, climate change, invasive species [12,18,19,20], or the interaction of several factors [18,21,22,23,24,25,26,27]. The importance and effectiveness of wild bee pollinators has increasingly been acknowledged in recent years [28]. These studies have emphasized the importance of a diverse community for pollination provision and ecosystem stability. Social bees such as honey bees and bumble bees are regularly used in pollination management, but only one genus of solitary bees is currently used in pollination management in European agriculture, the mason bees in the genus *Osmia* [29]. In addition, they represent an important group of wild pollinator species and this makes *Osmia* an interesting species to explore the effects of honey bee pathogens on solitary wild bees.

For honey bees, a variety of pathogens are known. Those comprise viruses, fungi, and bacteria. At least 23 viruses are known from honey bees [30] with several new viruses being discovered every year [31,32,33]. The ectoparasitic mite *Varroa destructor* and the viruses it transmits, particularly deformed wing virus (DWV), are considered the major cause of elevated honey bee mortality [34,35,36,37,38,39,40,41]. Over the last two decades, the emerging microsporidium *Nosema ceranae* has also become prevalent globally and has been shown to cause honey bee mortality [42,43,44], although the extent to which it is responsible for colony loss has been debated [45,46]. More recently, the wider risk posed by *N. ceranae* to sympatric wild bee populations has been explored, with studies revealing transmission of the parasite to bumble bees [47,48,49] and solitary bees such as *Osmia bicornis* [50]. This indicates frequent transmission potential to a broad range of bee hosts [51].

Pathogen detection alone does not provide information about consequences for host fitness. Defense against pathogens is usually energetically costly because of the upregulation of the immune system of the host resulting in physiological trade-offs [52,53,54,55,56]. Studying life history effects involves assessing their impacts on physiological states like mortality rates or tradeoffs [57].

Physiological states can reveal physiological costs due to an activated immune system. However, the costs can also depend on the host life stage.

Here, we exposed *O. bicornis* larva experimentally to *N. ceranae* spores to investigate if this exposure impacts physiological states. To our knowledge, no such experiments in solitary wild bees with *N. ceranae* have been conducted and nothing is known about life-history effects of this pathogen on solitary bees.

We addressed the following questions: (I) Does exposure with *N. ceranae* cause detectable establishment of the pathogenin the larval stage of *O. bicornis*? (II) Does exposure with *N. ceranae* affect development? and (III) Does exposure with *N. ceranae* impact the physiological state of the imago?

## 2. Materials and Methods

### 2.1. Study Species

*O. bicornis* is a common solitary wild bee species in Germany with a univoltine lifestyle and a flight period from March to June. The species uses wood cavities or crevices in buildings for nesting [58,59]. *O. bicornis* is polylectic and favors pollen resources close to the nest site [60,61]. Polylectic bees are forage on a wide variety of unrelated plants. For our experiment we ordered cocoons of *O. bicornis* from a breeder (BIENENHOTEL at www.bienenhotel.de).

### 2.2. Ethical Statement

Permissions for the study were provided by the Senate Department for Urban Development and the Environment of Berlin and included the allowance to release *Osmia bicornis* bees bought from the breeder according to § 40 subsection 4 BNatSchG. Moreover, permission according to § 45 subsection sentence 1 No. 3, sentence 2 BNatSchG for scientific reasons, and permission to catch and kill the bees according to § 44 subsection 1 No. 1 BNatSchG (ibid.) was granted.

### 2.3. Preparation of Spore Suspension for Inoculation

For our inoculation suspension, fresh *Nosema* spores from *Apis mellifera* were required. We collected samples from several hives in and around Berlin and investigated pathogen presence. A gut of a bee was dissected and homogenized in 200 mL of NaCl (0.9%). One drop was investigated microscopically (400×). If spores were present, the solution was processed with the DNeasy Plant Mini Kit from Qiagen (Hilden, Germany) according to the manufacturer’s instructions with the following modifications: 4 µL proteinase K was added with RNAse and heated incubation was extended to 30 min. We added 3–5 metal beads to the samples and disrupted them 3 times for 30 sec at full speed during the incubation period. For the final suspension, we used 30 µL of AE buffer.

For PCR, 5 µL DNA of each sample were mixed with 5 µL RNAse free H_2_O, 12.5 µL KAPA 2G Fast ReadyMix with dye (Kapabiosystems Roche Diagnostics, Mannheim, Germany) and 2.5 µL of the primers designed by Gisder & Genersch [62] and ordered from Metabion International AG (Planegg, Germany) applying the described PCR program. The amplified products were analyzed in a 1.5% agarose gel stained with SYBR Gold nucleic acid stain (Thermo Fisher Scientific, Darmstadt, Germany) and run for 80 min at 80 V. In each run, a positive control with confirmed *N. ceranae* bands was included as well as a negative control with ddH_2_O. Differentiation of the samples was based on presence of a 662 bp band [62]. A subset of the positive samples was sequenced by GATC Biotech and analyzed with BLAST to confirm the identity of the pathogen. If *N. ceranae* was detected, we chose the honey bees from one colony as spore source for the exposure experiment.

Fresh *N. ceranae* spore suspension for use in exposure experiments were maintained in laboratory kept groups of *A. mellifera* workers. Honey bees were kept according to Williams et al. [63]. After hatching, young honey bees were maintained in sterilized cages in groups of 50–200 individuals in a breeding chamber at 28 °C. For the inoculum, we used samples from one of the previously tested hives. We killed fresh bee samples by hand and immediately processed them according to Fries et al. [64] as described before. Ten days post-exposure, up to three bees were killed and processed further by the modified protocol of Fries et al. [64]. The bee gut was homogenized in 300 µL 1 5mM 9.0 pH buffered ammonium chloride (NH_4_Cl), and centrifuged (Eppendorf centrifuge 5810 R) at 5000 G (relative centrifugal force) for five minutes. The supernatant was discarded and the pellet was resuspended with another 500 µL NH_4_Cl by vortexing and filtering again. Spores were checked under a microscope with ×400 magnification, counted with a hemocytometer (improved Neubauer chamber) [65] according to Cantwell [66], and molecularly confirmed for species identity as described before. The positive tested suspensions were diluted with a 50% sucrose solution (w/w) to feed honey bee workers for infection. The inocula was diluted with ddH_2_O and applied to each larvae of *O. bicornis* aged 2 or 3 days with a concentration of 10,000 viable spores per bee and the same amount of autoclaved spores for the controls. The spore dose was based on guidelines derived from previous infection experiments [67,68,69,70].

### 2.4. Study Site and Experimental Set-Up

The study site was a 14 ha orchard meadow in Brandenburg, 32 km south of Berlin within the FFH-area Nuthe-Nieplitz-Niederung (N52°21′59.6″, E13°07′53.4″). Eighteen honey bee colonies from a beekeeper were located within a 250 m distance to the nest box. The hives had been at this location for several years. A nest box measuring 1.90 × 1.00 × 0.80 m was constructed. As nests, 60 nest boards accommodating 10 nests each were used with an acrylic, high temperature resistant cover to allow experimental manipulation. The diameter of the entrance was 8 mm, suitable for *Osmia bicornis* (www.bienenhotel.de, 01.04.2016). Bee cocoons and nest boards were ordered on www.bienenhotel.de and kept in 5 °C until placement in the nest boxes in the field on the 29th of April 2016.

One characteristic for sex determination in *O. bicornis* is body size with females being generally bigger and heavier than males [71]. We estimated sex via cocoon size. In order to avoid a low return rate of released bees, the recommended 1:1 sex ratio by the breeder was increased to 1.14:1 f:m resulting in an estimated 800 female and 700 male cocoons being placed in the nest boxes.

Bees were observed building brood cells and laying eggs. The hatching date was recorded in order to determine the age of the sampled larvae. Parasites, such as the parasitoids *Anthrax anthrax* (Schrank, 1781) and *Monodontomerus obsoletus* (Fabricius) or the parasitic fly *Cacoxenus indagator* (Loew), were cleared and brood cells with parasites were discarded from the experiment. Temperature and weather conditions were also recorded.

### 2.5. Treatment with N. ceranae

One µL of the viable *N. ceranae* spore-containing solution was placed either on the food source (treatment SpF) (N = 229) or directly on the larvae (treatment SpL) (N = 329). Corresponding controls were treated with solutions with autoclaved spore treatments termed CoF (N = 321) and CoL (N = 206), respectively. Food consisted of pollen in the larval brood chamber provided by the mother bee. For SpF and CoF treatments, a droplet of spore suspension was applied on the food directly in front of the larvae. For SpL and CoL treatments, the suspension was directly applied on the larval body on the first segments next to the head capsule. The randomized treatments of larvae were conducted between 13 and 29 May 2016.

### 2.6. Sampling of Osmia Bee Larvae

Finished brood cells were defined as closed cells with pollen provided and an attached egg and recorded daily between 14 and 29 May (except 18.5., 21.5., 26.5.) The hatching date was observed in order to determine larval age for inoculation for most of the hatched bees.

Between 5 and 16 June, 150 *O. bicornis* larvae were sampled on the 19th or 20th day post exposure, which corresponds to the 4th larval instar (22–24-days old). From a total of 150 larvae, 12 larvae were discarded due to presence of parasites in the brood cell or death. The sampled larvae were transferred individually to 1.5 mL microcentrifuge tubes and immediately frozen at −20 °C for measurements and dissection.

#### Physiological States of Osmia Bee Larvae

Head capsule width and fresh body weight of the hatched bee larvae (age 22–24 days) were taken as size measures.

The head capsule width was measured on the widest point with a binocular microscope (Olympus SZX16, ColorView, Olympus) with a caliper (1/50 mm nonius) according to Vogelweith et al. [72]. Referring to Bosch and Vicens [73], the head width constitutes the best estimators of adult weight and provision weight in both sexes besides wing lengths. No pollen for the provision weight was collected due to infestation with a parasitic moth in August, when the bees were in the pupal stage. The analyzed developmental stages mentioned in the following include the larvae, pharate referring to the individual enclosed in the cocoon, and the imago referring to the hatched individual.

### 2.7. Sampling of Pharates

A total of 526 pharates were sampled as fully developed bees within the cocoon (155–157 days old). The cocoon was gently cleaned by hand with a dry brush to remove feces and debris and stored immediately in a 1.5 mL microcentrifuge tube.

#### Physiological States of the Pharate

After recording the weight of the pupae, the cocoon was gently opened with a scalpel. The weight of the pharate (without cocoon) was recorded. The sex was determined based on the color of the clypeus [71,74]. Afterwards, the imago was frozen at −80 °C for 12 min to death, the fresh body weight was recorded, and the gut was removed from the body. Both groups, the sampled larvae and the pharates, were further dissected for the microscopic examination of the presence of *N. ceranae* spores.

### 2.8. Hatching Record of Imago

In total, 72 control and viable-spore-treated individual pupae were stored at 4 °C over winter and placed outside in two separate cages on 13 April 2017. Night and day temperatures, as well as hatching, were recorded daily. Hatching took place between 3 and 20 May 2017. Remaining cocoons were observed until 29 May when the experiment ended. The unhatched cocoons were counted. The heads of the hatched individuals were frozen at −80 °C. The remaining bee body was stored at −20 °C.

#### Physiological State of the Imago

We used inter-tegular span as a measure of insect size as it is well correlated with flight range for bees [75]. We also measured fat body content as indicators of immunity and longevity. The fat body stores energy and synthetizes immunoproteins [76].

Fat body content was measured using methods described by Mikolajewski et al. [77] and De Block et al. [78]. The bodies of all hatched individuals were weighed and subsequently dried in an incubator at 48 °C. Afterwards, 1.5 mL of dichlormethane was added. Samples were put on a shaker for 24 hours after which the dichlormethane was removed and the bees weighed again. Fat body mass was determined by substraction.

Body size and flight muscle ratio were measured as described by Plaistow et al. [79]. The dry fatless thorax was placed in 0.2 M potassium hydroxide at room temperature for 48 h which results in digestion of the flight muscle. Afterwards, the remaining cuticle was washed in distilled water, dried, and re-weighed. Flight muscle ratio was determined as the quotient of dry flight muscle mass and total dry abdomen and thorax mass.

### 2.9. Detection of N. ceranae in O. bicornis in Larvae and Pharate

Larvae (N = 138) were processed either as whole body or separated guts only (Appendix A Table A2). The guts of pharates (N = 507) were dissected as described for honey bees [64]. The guts of the two developmental stages were processed according to Fries et al. [64] as described above with the following modifications. The gut was mixed with 300 µL sterile ddH_2_O for homogenization and centrifugation. The supernatant was discarded, and the pellet was resuspended with 300 µL sterile ddH_2_O and filtrated once through cotton wool. Gut suspensions and remaining pharate bodies were frozen.

After spore preparation, presence of *N. ceranae* spores was checked with a phase contrast microscope (400×). If spores were present, the solution was processed with the DNeasy Plant Mini Kit from Qiagen (Hilden, Germany) as described above.

### 2.10. Statistical Analysis

All statistical analyses were conducted in R 3.3.4. Data were tested for normal distribution of residuals (larva, pharate) and homogeneity of variance or Shapiro-test (imago) and log-transformed if necessary. Where residuals were normally distributed, the data was tested with linear models. The gaussian family was applied for all models.

#### 2.10.1. Statistical Analysis in Larvae and Pharates

Differences in pupation start and head capsule width were fitted against the explanatory variable “treatment” (viable-spore exposed/control). A linear multi factorial model was also fitted to the data with one-way ANOVA. The ‘pupation start’ (in days) as a response variable was tested, using treatment as a fixed factor and head capsule width as a co-variable. For the larval dataset, ANOVA was applied using head capsule width as the response variable and using treatment as a fixed factor.

Furthermore, larvae as well as the pharate dataset were tested with ANCOVA with head capsule width as response, weight difference between pupae and cocoon as fixed factor, and sex as co-variable. For the pharate dataset, weight difference as a response was tested against treatment as a fixed factor and sex as a co-variable.

Finally, linear regressions were carried out in the larval and the imago datasets by fitting head capsule width against fresh weight or pupal weight, respectively. Another regression analysis was performed to inspect the relationship between head capsule width and fresh body weight in both sexes.

#### 2.10.2. Statistical Analysis of Imaginal Physiological State

Differences between groups were tested using general linear models. The analysis of the hatching rate included all 144 samples. For analysis of physiological states, 11 individuals were excluded from the dataset due to measurement erros resulting in N = 133.

## 3. Results

From a total of 1592 recorded brood cells, 1085 *O. bicornis* were treated with either viable or autoclaved *N. ceranae* spores, of which a total of 783 (138 larvae; 512 pharates; 133 imagines) *Osmia* bees were sampled for use in analyzing physiological state/developmental statistics, respectively (Table 1). For analyzing mortality from 1085 bees, a sample set of 915 was used. The sex ratio of sampled pharates was calculated from 506 individuals from the pharate dataset (140 female, 366 male). The overall sex ratio for all sampled pharates was 1:2.6 (female:male), including those bees which were parasitized but sex could still be determined from the remains of the head capsule. Of the imago dataset, 67 and 66 hatched imagines in control and viable spore-treated groups were analysed for differences in physiological state.. The spore application method showed no significant effect in the statistical models.

### 3.1. Mortality of Infected O. bicornis Larvae and Pharates

From 1085 treated bees, 176 bees died for reasons unrelated to experimental treatment, including predation or parasitism (N = 88). The remaining 88 bees that died during the semi-field experiment were derived from the following treatments; CoF (control on food): N = 22, CoL (control on larvae): N = 14, SpF (spores on food): N = 18, SpL (spores on larvae): N = 34. Nine pharates from the sampled bees were detected as dead when sampled (opened the cocoon). Of the 88 bees, 80 could be assigned to the development stages larvae (69) and pharates (11) with CoF, N = 20, CoL, N = 9, SpF, N = 18, and SpL, N = 33.

**Figure 1:**
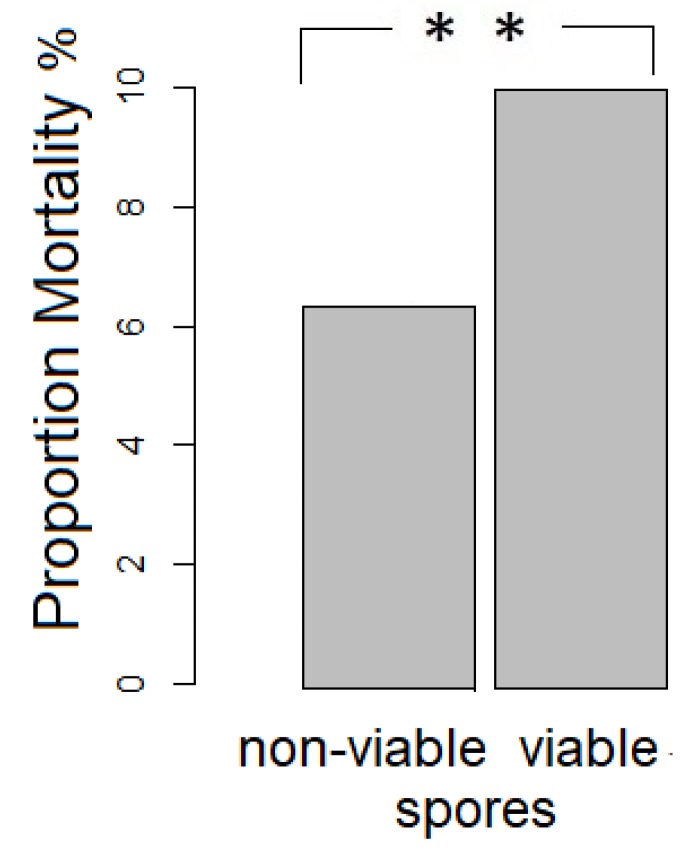
Significant differences in mortality between treatment groups including both larval and pharate state (N = 915, deaths non-viable spore exposure: 29, deaths viable spore exposure: 51).

A Pearson‘s Chi-squared test with Yates‘ continuity correction revealed that significantly fewer bees from the control vs. viable spore treatment died when considering both developmental stages (ꭓ-squared = 5.2176, df = 1, *p* = 0.02236, Figure 1). Within the larval stage fewer bees from the control versus the viable spore treatment had died (ꭓ-squared = 4.3825, df = 1, *p* = 0.03136), whereas there were no significant differences in the pharate state (ꭓ-squared = 4.3825, df = 1, *p* = 0.5859).

### 3.2. Detection of N. ceranae Spores

From 545 samples (larvae and pharate), 334 were treated with viable *N. ceranae* spores. A total of 1.5% (5 bees) of the viable spore treated individuals yielded positive homogenates (positive detection of *N. ceranae* spores) with <21 spores per 10 µL gut homogenate each.

**Figure 2:**
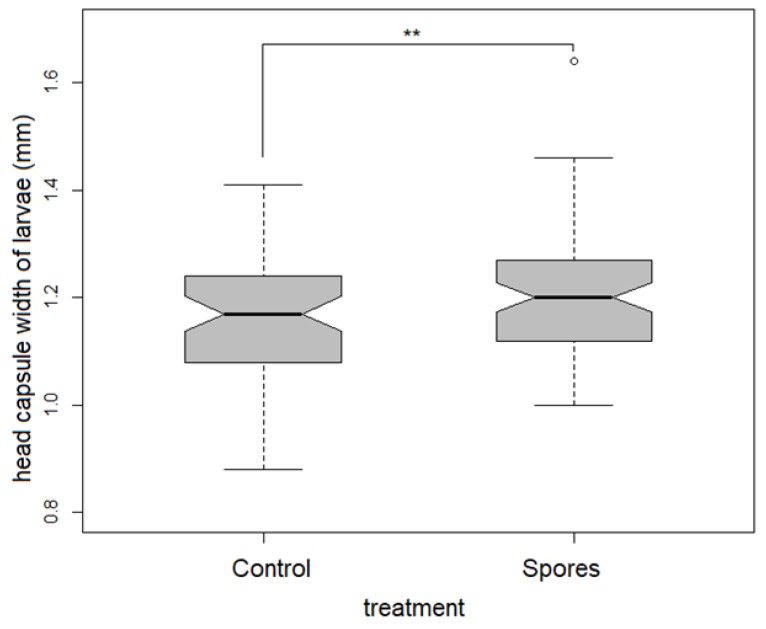
Significant differences in head capsule width in treatment groups within the larvae of both sexes.

Physiological state: Head capsule width was smallest in the control treatment, both in the larval as well as the pharate dataset. ANCOVA revealed a significantly higher mean head capsule width in the viable spore treated groups in the larval dataset (F_1,133_ = 6.632, *p* = 0.011, Table 2, Figure 2).

The body size of the pharate revealed a strong correlation between head capsule width and fresh bee weight (r = 0.782). The strongest positive correlation was detected between cocoon weight and fresh weight (r = 0.995). The head capsule width, pupal weight, and fresh body weight of female pharates was 1.5 times that of males (Table A1, Table A2 and Table A3). The proportion of females in the ‘Spore’ group was 32.2% and 24% in the ‘Control’ group. The proportions differed significantly (ꭓ-squared: 4.808, df = 1, *p* = 00283).

**Figure A1:**
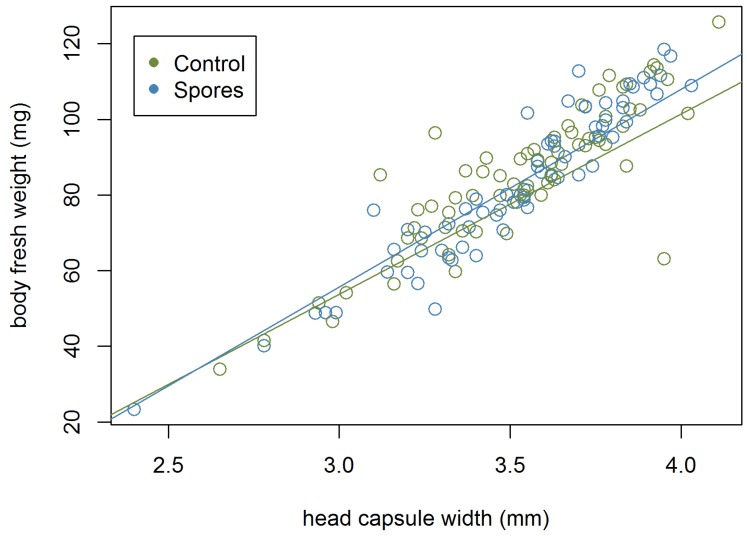
Female head capsule width and fresh body weight (pharate dataset), N = 139.

**Figure A2:**
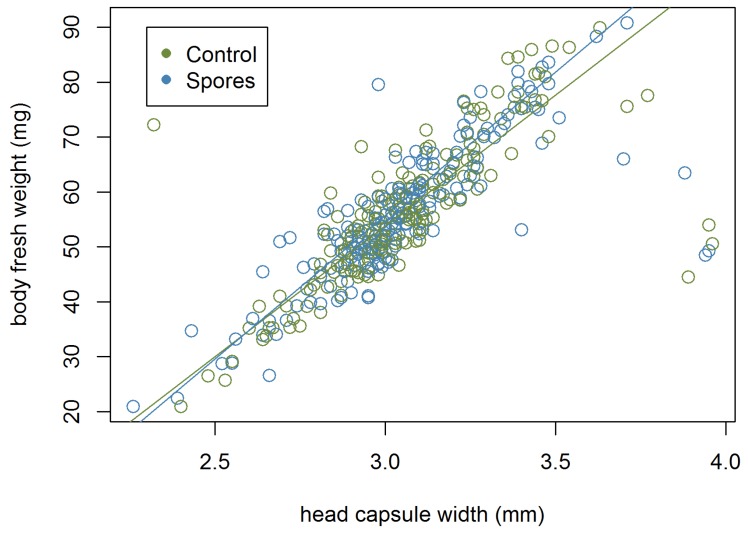
Male head capsule width and fresh body weight (pharate dataset), N = 363.

An analysis of covariance of head capsule width and fresh weight (F_2,499_ = 950.1, *p* < 0.001, Table 2) as well as head capsule width and pupal weight (F_2,499_ = 956.4, *p* < 0.001, Table 2) with sex as co-variate revealed significant differences between sexes. Regressions of head capsule width to fresh body weight for both sexes are shown in Figure A1 and Figure A2.

### 3.3. Development Time of the Larvae

**Figure 3:**
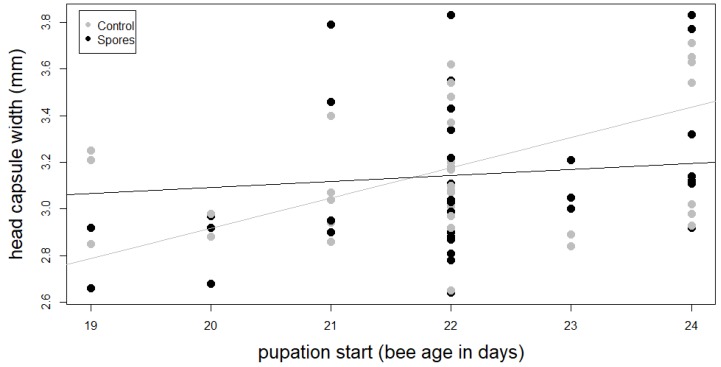
Interaction between head capsule width and pupation start of main treatment groups ‘Control’ and ‘Spores’.

The ANOVA of the transformed linear model revealed a significant interaction between treatment and head capsule width on the onset of pupation (F_1,74_ = 4.61, *p* = 0.035, Table 2, Figure 3, Table A4).

### 3.4. Physiological State Imago

Adult emergence rate was 93% (sterile spore) and 91.6% (viable spore), N=144. Sterile and viable spore treated groups for the subsequent physiological state analysis contained 26 females and 41 males, and 26 females and 40 males, respectively as individuals with measurement errors were excluded.

Fat content was compared between treated and control males and females. As the data was not normally distributed (males: Shapiro-Test *p* = 0.03255, females: (Shapiro-Test *p* = 0.03404).) a general linear model was performed that showed no significant differences in fat content between the treatments in males (F_79,81_ = 0.257, *p* = 0.798) or females (F_36,52_ = 1.72, *p* = 0.0941). For tegulae size, the data were normally distributed in males (Shapiro-Test *p* = 0.3696) and females (Shapiro-Test *p* = 0.5313), but as above with fat body, a linear model indicated no significant difference between the groups (males: F_79,81_ = 0.4379; females: F_36,52_ = −1558, *p* = 0.1281). The wing muscle weight analysis showed no significant difference in males (Shapiro-Test *p* = 0.01899, glm: F_76,78_ = 1360, *p* = 0.178) or females (Shapiro-Test *p* = 1.03 × 10^−4^, glm F_37,39_ = 0.994, *p* = 0.32667, Table 3).

## 4. Discussion

In our study on a solitary bee exposed to a honeybee pathogen during development, we found treatment-dependent life history responses in different physiological states. We detected (1) significantly higher mortality in viable spore-exposed individuals and low spore detection as well as (2) a delayed onset of pupation related to an interactive effect of increased head capsule width and spore-exposure. However, (3) no impact on physiological states of hatched individuals were found.

(1) The higher mortality in the viable spore-treated larvae and pharates could be explained by the high effort of synthesis of storage proteins during larval development. As has been demonstrated in honey bees, triggering a stronger larval immune response can result in lower spore load in adults but can impose a cost in the form of reduced life-span [70]. Moreover, in *A. mellifera*, *N. ceranae* impairs its host by damaging intestinal tissue and preventing renewal by inhibiting defense genes [80], using host-ATP for its own growth and reproduction [81]. It is also thought to suppress the host’s immune response [82,83,84,85]. The deprived hosts consequently lack functional tissue, energy necessary for its own metabolic maintenance and can invest less in immune defense. A combination of these processes may explain our findings. For example, in starved bumble bee workers, immune challenge resulted in decreased survival [54]. Furthermore, it has been shown that short-term survival is traded off in order to ensure reproductive success in an ant queen [86].

We found only very few individuals with spores in the cohorts treated with viable spores. The results contrast with infection experiments with *N. ceranae* on honey bee larvae [70] and *Nosema bombi* in larvae of *Bombus* spp. [87]. Differences to other studies might be attributed to initial spore doses, bee species, strains of *Nosema*, the bee immune responses at different developmental stages, or combinations of these factors [88,89]. We used the same dose of the same species as in one cohort in Eiri et al. [70] where it resulted in spore elevations in larvae in *A. mellifera*. Our low spore detection in the larvae taken out of the brood cells suggests no establishment of the pathogen. It was demonstrated in honey bees that some individuals exhibited resistance to the pathogen by countering its manipulation of apoptosis and defecating the infected cells [90].

(2) Immune activation and maintenance in infected insect hosts require high nutritional and energetic resources which then cannot be shunted into growth and development [52,91,92,93,94,95]. Thus, we assessed the impact of a treatment with viable spores on the physiological state of growth and development times. The literature results [52,91,92,93,94,95] contrast with our finding of an increased head capsule width in viable-spore-treated individuals. However, an infection with *N. ceranae* increases hunger level, thus food intake, and could present an explanation [96]. The delayed start of pupation we observed in the viable-spore-cohort with an increased head capsule width could be attributed to a trade-off between investments in defense versus development. Increased pupation periods after immune challenge were reported in a variety of studies such as in the beet armyworm [97], the tobacco caterpillar [98], and cabbage loopers [99].

(3) We found no difference in fat content between viable-spore-treated and control individuals in the hatched imagines. Apart from energy storage, the fat body has an essential role in the immunity of insects. In response to an immune challenge, lipids are mobilized to the haemolyph [100,101]. Lipids are suggested to be used as an energy resource in combating the infection or in membrane biogenesis in haemocytes [102]. Consequently, variations in fat body mass might impact effectiveness of immune response. The present study suggests that exposure with viable *Nosema* spores does not result in impacts on the investigated physiological state parameters of hatched individuals. Body size, measured through tegulae distance, did not differ either. Body size is determined by conditions during larval development [103]. All bees were provided with an adequate amount of pollen. Our results suggest that the cost of immune defense was paid earlier during development, consistent with the higher mortality in spore-treated larvae.

## 5. Conclusions

Treatment with viable spores of *N. ceranae* resulted in higher mortality during the larval stage but spores were only detected in low numbers in a few of the exposed individuals indicating that the pathogen cannot easily establish itself in this species. An interaction between treatment with viable spores and increased head capsule width resulted in delayed pupation start which might be due to immune effects during development, although no impacts on physiological states could be detected in the hatched imagines. Even in the absence of successful infection, exposure to *N. ceranae* could still compromise the development and life history of *O. bicornis*.

## Figures and Tables

**Table 1:** Overview of study sampling.

Samples	Sample Size Registered Brood Cells (from N = 1592)	Sample Size for Mortality Rates (from N = 1085)	Data Set Size for Statistic (from N = 1085)	Head Capsule Width/Weight	Spore Check
All bees	1085	915	783	645	545
Larvae			138	138	138
Pharates			512	507	407
Imagines			133	-	-

**Table 2:** Results of a linear multi-factorial model exploring the effect of treatment (viable vs. sterile spores) on the pupation start, head capsule width, and weight differences during development and results of regressions of the three datasets for two development stages. Head capsule width, bee age, or sex were used as covariates (C). lm = linear model. A table with estimates can be found in the Appendix A (Table A5).

Samples	Depen-dent Variable	Independent Variable	Co-Variate (C)/Interaction (I)	Num. d.f.	F-value	*p*-Value	Analysis
pharate (history)	pupation start	treatment, inoculation type	C: head capsule width	73	4.304	0.0035	lm: anova
pharate (history)	pupation start	treatment	I: head capsule width	74	6.521	0.0005	lm: anova
larva	head capsule width	treatment		133	6.632	0.011	lm: anova
pharate	head capsule width	fresh weight	C: sex	499	950.1	<0.001	regression
pharate	head capsule width	cocoon weight	C: sex	499	956.4	<0.001	regression
pharate	Fresh weight	cocoon weight	C: sex	499	6.292e+04	<0.001	regression
pharate	weight difference	treatment	C: sex	500	3.934	0.0479	lm: anova

**Table 3:** Physiological state tests of hatched imagines (males/females) from spore-treated and control groups.

Dependent Variable	Independent Variable	Shapiro-Test (*p*-Value)	Model	Residual Deviance	Num. d.f.	F-Value/ t-Value	*p*-Value
fatbody weight	males s/c	0.03255	glm	2753.3	79	0.257	0.798
fatbody weight	females s/c	0.03404	glm	0.0025140	36	(−)1.72	0.0941
tegulae distance	males s/c	0.3696	lm		79	0.4379	0.5101
tegulae distance	females s/c	0.5313	lm		36	(−)1558	0.1281
wing muscle weight	males s/c	0.01899	glm	0.00036717	76	(−)1360	0.178
wing muscle weight	females s/c	1.03 × 10^−4^	glm	0.00055552	37	0.994	0.32667

## Appendix A

**Table A1:** Overview for processed larvae and pharates during sampling.

Sample size	Treatment	Count of Processed Part of the Larva for Spore Check	
Sum of larvae		whole larvae	separated larvae (body and gut)
61	Control on food	15	18
	Control on larva	20	8
77	Spores on food	35	2
	Spores on larva	21	19
Sum of pharate		separated pharate (gut separated for spore check, spore check with n = 407)	
255	Control on food	127	
	Control on larva	128	
257	Spores on food	129	
	Spores on larva	128	

**Table A2:** Measurements dependent on sex within the pharate dataset.

Sex	Kind of Measurement	N	Mean	SD	Min	Max
**females**	head capsule width	139	3.52	0.30	2.40	4.11
	pupal weight (mg)		97.33	23.16	25.98	148.10
	fresh body weight		84.22	19.31	23.29	125.8
**males**	head capsule width	363	3.05	0.26	2.26	3.96
	pupal weight (mg)		66.21	15.44	23.03	108.75
	fresh body weight		56.28	21.155	20.89	90.83

**Table A3:** Overview of measurements dependent on sex within the pharate dataset.

Sex	Kind of Measurement	N	Mean	SD	Min	Max
**females**	head capsule width ‘Co’	59	3.54	0.28	2.40	4.03
	head capsule width ‘Sp’	80	3.51	0.32	2.65	4.11
	pupal weight ‘Co’	59	97.59	21.91	25.98	131.12
	pupal weight ‘Sp’	80	97.13	24.18	37.83	148.10
	fresh body weight ‘Co’	59	84.23	18.38	23.29	112.81
	fresh body weight ‘Sp’	80	84.22	20.08	34.01	125.80
**males**	head capsule width ‘Co’	193	3.06	0.27	2.26	3.96
	head capsule width ‘Sp’	170	3.05	0.24	2.32	3.89
	pupal weight ‘Co’	193	64.78	15.10	23.03	108.75
	pupal weight ‘Sp’	170	67.83	15.70	25.89	102.14
	fresh body weight ‘Co’	193	55.18	12.32	20.89	90.83
	fresh body weight ‘Sp’	170	57.53	12.78	22.43	85.92

**Table A4:** Dispersion of dataset history for the set of pupation start.

**Treatment**	**Dataset/Treatment**	**N**	**Mean**	**SD**	**Min**	**Max**
	Complete dataset	87	22.0	1.4	19	24
Control	Control on food	24	22.1	1.4	19	24
	Control on larva	23	21.5	1.3	19	24
	Control sum	47	21.8	1.4	19	24
Spores	Spores on food	28	22.5	1.5	19	24
	Spores on larva	12	21.8	0.5	21	22
	Spores sum	40	22.3	1.3	19	24

**Table A5:** Results of a linear multi factorial model exploring the effect of treatment (Viable vs Sterile spores) on the pupation start, head capsule width and weight differences during development and results of regressions of the three datasets for two development stages. Head capsule width, bee age or sex were used as covariates (C). lm = lineaer model, Estimate abbreviations: c.weight = cocoon weight, head = head capsule width, m = male Sp = Spores.

Samples	Depen-Dent Variable	Independent Variable	Co-variate (C)/Interaction (I)	Num. d. f.	F-Value	*p*-Value	Estimate
pharate (history)	pupation start	treatment, inoculation type	C: head Capsule width	73	4.304	0.0035	Head 1.44769
pharate (history)	pupation start	treatment	I: head Capsule width	74	6.521	0.0005	treatSp 6.8983 Head 2.5875 treatSp:head −2.0463
larvae	head capsule width	treatment		133	6.632	0.011	treatSp 0.05150
pharate	head capsule width	fresh weight	C: sex	499	950.1	< 0.001	Weight 0.0149293 Sexm −0.0552998
pharate	head capsule width	cocoon weight	C: sex	499	956.4	< 0.001	c.weight 0.0123092 sexm −0.0894422
pharate	Fresh weight	cocoon weight	C: sex	499	6.292e+04	< 0.001	c.weight 0.82060 sexm −2.40835
pharate	weight difference	treatment	C: sex	500	3.934	0.0479	teatmentSp 0.6557
